# A Novel Scientometrics Research on the Interaction between Oxidative Stress and Hematopoietic Stem Cell Transplantation Complications: From Graft-versus-Host Disease to Sepsis

**DOI:** 10.1155/2023/7708085

**Published:** 2023-01-27

**Authors:** Shaokang Xu, María José Cavagnaro, Jian Shi

**Affiliations:** ^1^Department of Hematology and Critical Care Medicine, The Third Xiangya Hospital, Central South University, Changsha, China; ^2^College of Medicine-Phoenix, The University of Arizona, Phoenix, Arizona, USA

## Abstract

As major and serious complications after hematopoietic stem cell transplantation (HSCT), graft-versus-host disease (GVHD) and sepsis are the chief causes of low survival rates as well as mortality and for HSCT recipients. Although the overall treatment outcomes of HSCT have improved significantly in recent years, there is still an increased incidence rate of complications and mortality after transplantation. In the immediate past, with a deeper understanding of oxidative stress, more and more shreds of evidence have shown that it is closely related to transplantation-related sepsis. However, there is currently a precious little research on the interaction between oxidative stress and complications after HSCT, and the major mechanism has not yet been clarified. The objective of this study was to assess the internal connection between and potential mechanisms as well as visualized the scientometrics results of all important literature related to the topic. Through exhaustive scientometrics analysis, we searched and carefully screened 286 related publications from the Web of Science Core Collection (WoSCC) with “((HSCT) OR (hematopoietic stem cell transplantation)) AND (oxidative stress)” as the search strategy. Then, detailed visualization of the overall information analysis was made by scientific and rigorous bibliometrics software or website. Next, we analyzed retrieved articles extensively and then 59 publications that are relevant to this topic were selected for nuanced analysis and summary. The assessment of these studies proved the validity of the interaction between oxidative stress and complications after HSCT objectively and directly.

## 1. Introduction

Hematopoietic stem cell transplantation (HSCT) refers to a treatment method that pretreats the transplant recipient through high-dose radiotherapy, chemotherapy, or other immunosuppressants, clears the tumor cells and abnormal clonal cells in the recipient, blocks the pathogenesis, and then transfuses autologous or allogeneic hematopoietic stem cells to the recipient so that the recipient can reestablish normal hematopoietic and immune functions in the body, so as to achieve the purpose of treatment [1–3]. Hematopoietic stem cell transplantation is widely used in the treatment of malignant hematological diseases, nonmalignant refractory hematological diseases, some solid tumors, genetic diseases, epidemic diseases, and congenital metabolic diseases and has achieved good curative effects. Although HSCT has a better curative effect and more and more applications, it also has many serious complications. It includes infection, transplant failure, graft-versus-host disease (GVHD), and hepatic vein occlusion disease (VOD) [4, 5]. One of the most common factors that affect the long-term survival of transplant individuals is infection and its related sepsis [6].

Sepsis is a clinical syndrome caused by excessive inflammation after infection, including immune abnormalities, coagulation abnormalities, and systemic multiple organ dysfunction [7]. The early manifestation of sepsis is a systemic inflammatory reaction. After receiving hematopoietic stem cell transplantation, patients have a significantly increased chance of infection-related sepsis due to the suppression of immune function. Once sepsis occurs, it gradually turns into shock, multiple organ failure, and eventually death with the development of the disease [8, 9]. The disease progresses rapidly, the prognosis is dangerous, and the clinical treatment is complex. It has become one of the main causes of treatment failure and death after hematopoietic stem cell transplantation. Some studies have pointed out that studies have shown that the increase of proinflammatory cytokines in the body and the process of oxidative stress caused by them are two of the basic pathological changes of sepsis, while patients with hematopoietic stem cell transplantation often have problems such as intestinal flora disorder, intestinal barrier function decline, and systemic defense function damage in the process of diagnosis and treatment, which will lead to an increased risk of infection from wide range pathogens, and its severity is closely related to mortality [6]. At the same time, although the international community has provided guidelines for the diagnosis and treatment of severe sepsis, the mortality of severe sepsis is still high, and clinical work is still facing great challenges. Although a series of studies have proved that oxidative stress (OS) plays a very important role in the pathological procedures of both sepsis and HSCT-related complications [10–12], solving sepsis related to hematopoietic stem cell transplantation is still a major problem that puzzles clinical and basic research, especially the in-depth study between oxidative stress and intestinal flora and the related scientometrics research and visual presentation, which also urgently needs to be studied in depth.

Scientometrics is a novel discipline that mainly focuses on the research of the quantitative angle of the scientific process as an important communication system [13]. Scientometrics focuses on the quantitative characteristics and characteristics of science and scientific research. The key and difficult point of it is how to conduct in-depth research and investigation on scientific development and mechanism through statistical mathematical methods. In recent years, scientometrics has played an important role in the performance evaluation and measurement of various scientific researches [14]. Scientometrics focuses on but is not limited to citation analysis in academic literature. It can also conduct qualitative and quantitative analyses of literature from multiple dimensions, such as cooccurrence analysis, cocitation analysis, coauthor analysis, and bibliographic coupling. Moreover, scientometrics usually involves various in-depth clustering analyses. In basic scientific metrology research, the most basic process and research foundation are to construct the citation map, which can show the detailed network of citations between different publications in charts [15–17]. In addition, visualization is also an important attribute and feature of scientometrics as well as bibliometrics [18]. By integrating complex and difficult-to-understand data and presenting them in the form of images, researchers can clearly and quickly obtain the latest research hot spots and directions, thus providing a theoretical basis for further research. Scientific metrology has become a popular research method in many fields based on large-scale literature databases. In this study, we searched and carefully screened all relevant publications in the science core collection network for comprehensive and accurate analysis and scientific measurement for further processing and scientific metrology research.

Therefore, the purpose of this study is to conduct real-time scientometrics analysis of existing relevant studies and present the latest trend of the interaction between oxidative stress in HSCT and its related sepsis, so as to find the current research hot spots, lay a theoretical foundation for subsequent clinical research, and further provide the relevant research foundation for the application of evidence-based medicine and translational medicine, with the aim of providing new insights for OS- and HSCT-related complications.

## 2. Materials and Methods

### 2.1. Search Strategies

A publication search was conducted in the Web of Science Core Collection (WoSCC) of the Web of Science database on July 19, 2022. Firstly, we used ((HSCT) OR (hematopoietic stem cell transplantation)) (All Fields) AND (oxidative stress) (All Fields) as the search strategy, and then, 286 publications were obtained. Next, we read all the search results extensively and excluded 227 publications that were not closely related to postoperative complications. Finally, 59 publications were obtained. Then, we exported all the full records and cited references with the data style of TXT.

### 2.2. Data Processing

In the process of data processing, VOSviewer (version 1.6.17) and Citespace (version 6.1.R2) and an online website of bibliometrics (https://bibliometric.com/) were used to conduct the following analysis and visualizations based on the scientometrics and bibliometric principle:

1. Cluster analysis and hot time presentation of all keywords from 286 publications based on the cooccurrence relationship
2. The timeline visualization of the keywords from the 59 screened publications
3. Coauthor relationship between countries and organizations and their relative number of publications
4. Statistical analysis of basic information of journals with the largest number of publications
5. The cluster analysis of the cited journals is based on the cocitation relationship
6. Visualization of citation relationship and cluster analysis between journals of the screened publications and their references

## 3. Results

### 3.1. Keyword Clustering Analysis of the First Search Results

**Figure 1:**
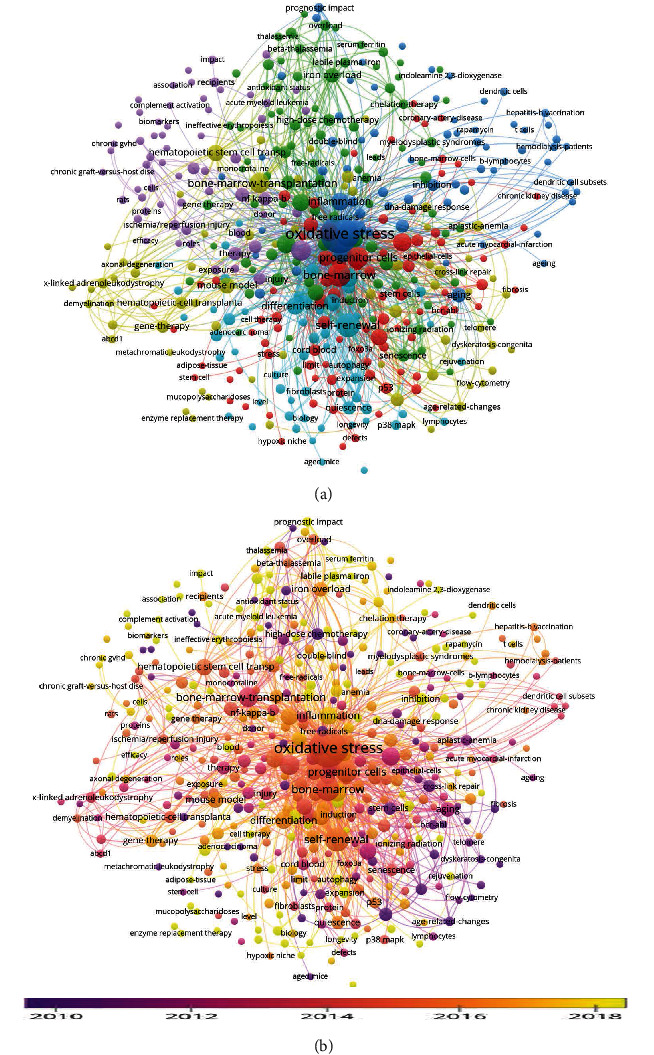
Keyword analysis based on initial search results. (a) Cluster analysis was performed on all keywords based on the cooccurrence relationship. Keywords of the same color come from the same cluster. The size of bubbles represents the frequency of keywords, and the connection between bubbles reflects the cooccurrence relationship between them. (b) Hot spot time analysis of keyword occurrence. Through analysis, the time point with the highest frequency of each keyword is obtained and this information is visualized. The corresponding relationship between color and different times can be seen in the legend.

After cluster analysis of 286 publications in the field of HSCT and oxidative stress using VOSviewer software, the cooccurrence relationship and occurrence frequency of keywords are presented (Figure 1(a)). Among the total 1638 keywords, 111 keywords have a frequency of occurrence equal to or more than 5 times. We present the occurrence and link strength of keywords with times of occurrence greater than 10 in Table 1. In addition, based on cluster analysis, we also analyzed the time nodes with a high frequency of occurrence of each keyword (Figure 1(b)).

### 3.2. In-Depth Keyword Clustering Analysis of Screened Publications

**Figure 2:**
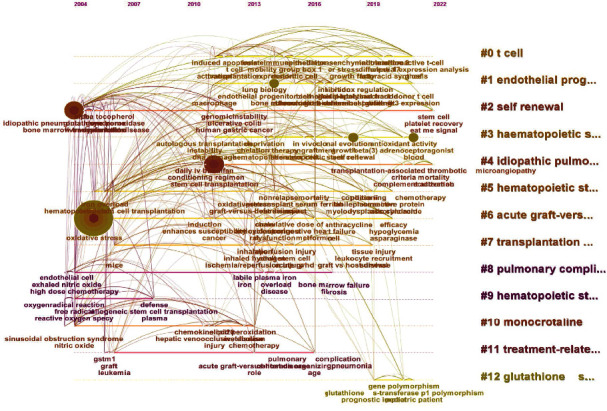
The timeline view of cluster analysis of keywords from screened publications.

After excluding studies unrelated to complications or prognosis of hematopoietic stem cell transplantation, we obtained 59 publications. After that, an in-depth analysis of the keywords of these publications using Citespace software (Figure 2) was also performed. Cluster analysis based on cooccurrence relationship is used here and 13 clusters are obtained. In addition, each cluster is marked with a label, and the keywords under the label are closely related to it. The temporal hot spots of the keywords are analyzed and presented in the figure, and the cooccurrence relationship between them is presented in the form of lines.

All the keywords were clustered based on the cooccurrence relationship, and 13 clusters were obtained. The labels of each cluster are displayed on the right side, and the time when the keywords mainly appear is also indicated on the horizontal axis. In addition, the connection between keywords also reflects their cooccurrence relationship.

### 3.3. Analysis of Coauthorship between Organizations and Countries

**Figure 3:**
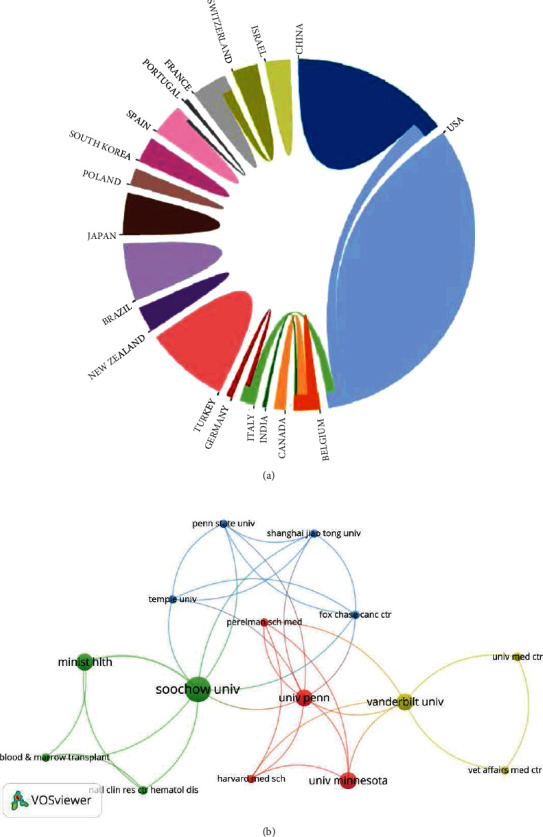
The coauthor relationship in screened publications. (a) Cooperation between countries. In the chord diagram, different color blocks are used to correspond to countries, and the area reflects the number of national publications. The connection between the color patches reflects the cooperative relationship between countries. (b) Cooperation between organizations. The bubble size reflects the number of publications, and the connection between them reflects the coauthor relationship between organizations. In addition, the clustering situation is also shown in color in the figure.

Here, we continue to conduct an in-depth analysis of the cooperation relationship and research situation among the units of 59 selected publications so as to find out the research units with deep research in this field. The online website of bibliometrics (https://bibliometric.com/). It is used for cooperation between countries (Figure 3(a)). Among them, the United States and China have the largest number of publications, and their cooperation is close. The cooperation relationship of the organization is completed by using VOSviewer software through clustering analysis based on the coauthor relationship (Figure 3(b)). We can intuitively see from the figure that Soochow University has a large number of publications, and the University of Pennsylvania has the largest number of cooperation projects with other institutions.

### 3.4. Association Analysis between Journals Based on Citation Relationships

**Figure 4:**
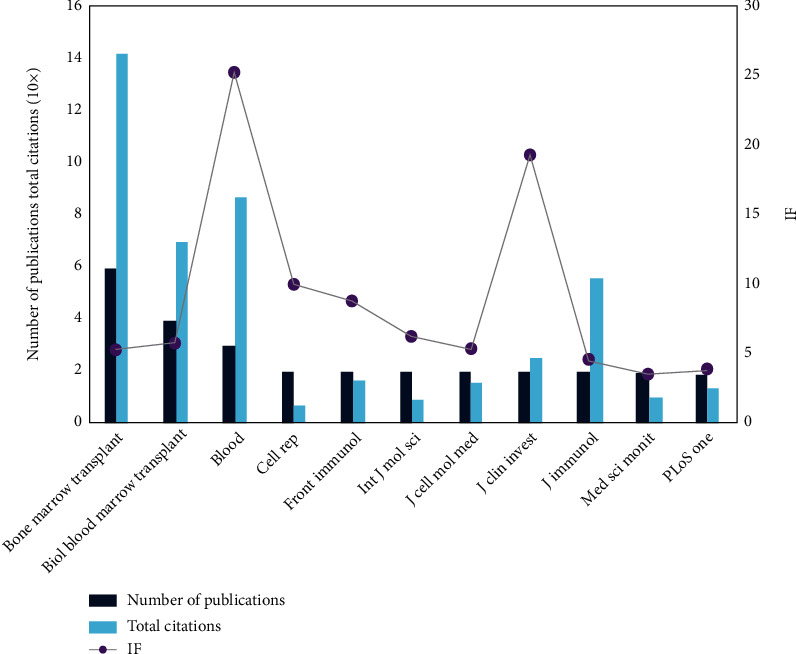
Basic information of journals with a top number of publications.

**Figure 5:**
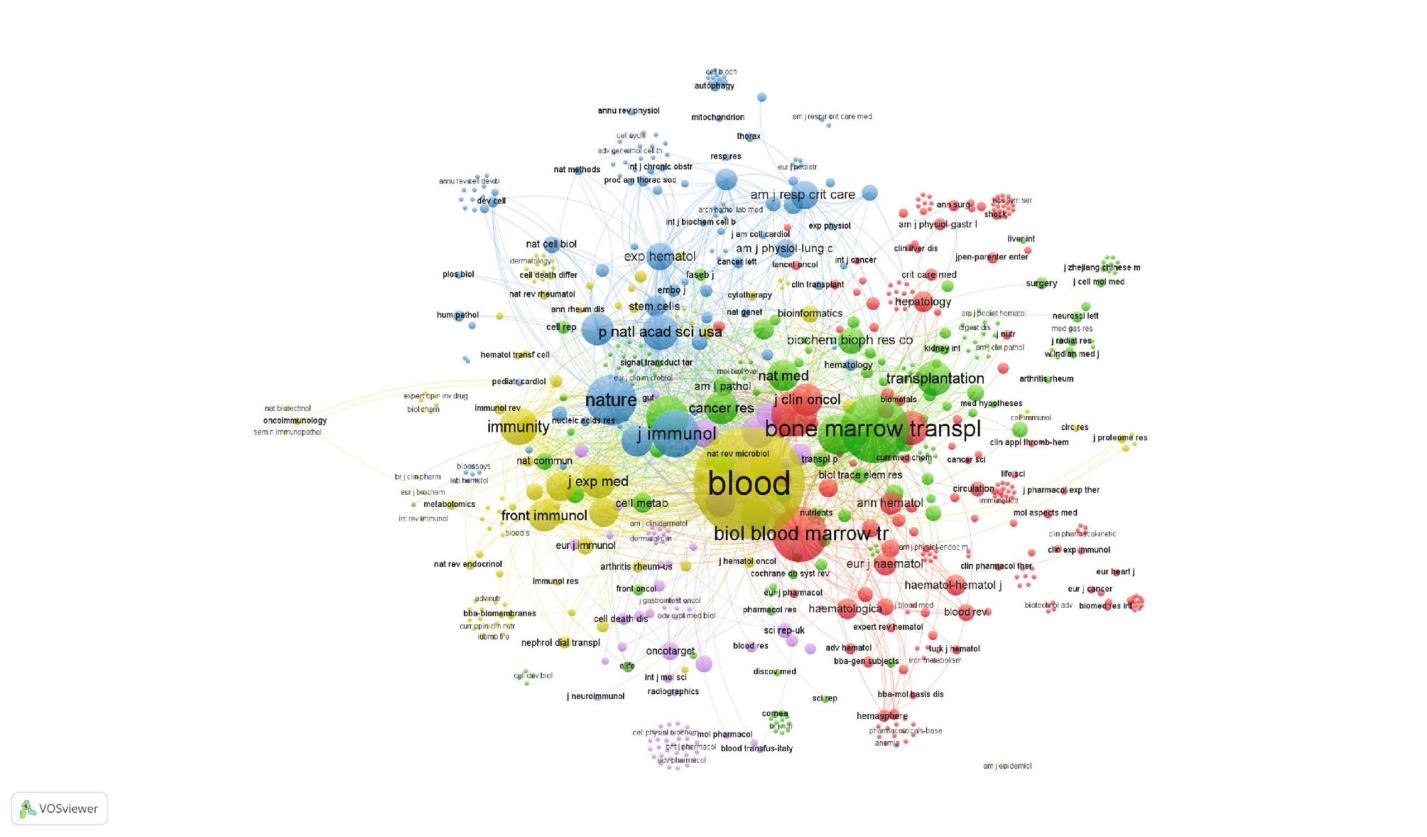
Cluster analysis of cited references of the screened publications based on the cocited relationship.

Firstly, we statistically processed the journals with more screened publications and visually presented the total number of publications, the total number of citations, and the impact factor (Figure 4). The number of publications, the total citation times, and the impact factor of the journals are presented. After that, for the references of these 59 publications, we conducted a cluster analysis based on the cocitation relationship for the journals from which they came (Figure 5). The connection reflects that they are cited by a publication at the same time, and the bubble size reflects the number of publications. In these two analyses, blood, bone marrow translation, and biology blood marrow translation all have high occurrence times and high if, which means they have a high influence in this field. Finally, the citation relationship between the screened publications and the journals from the reference sources is presented, and all the journals are clustered into multiple clusters, where the topic relevance is presented.

**Figure 6:**
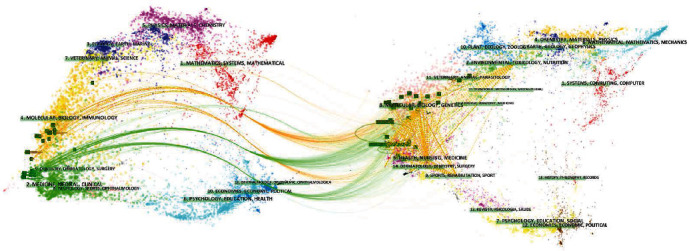
Visualization of citation relationship and cluster analysis between journals.

And then, the visualization of citation relationship and cluster analysis between journals have been made (Figure 6). The left part is the journal of our screened publication, and the right part is the journal of the reference of the publication. The journals on the left and right sides have done cluster analysis according to the citation situation (they have been clustered into several clusters, and the specific situation has been presented in the figure with labels). The middle line shows the main reference situation.

## 4. Discussions

One of the most common complications after hematopoietic stem cell transplantation is infection and its related sepsis because as long as large doses of radiotherapy and chemotherapy are carried out, there is a period of bone marrow suppression, which will cause patients to cause a variety of infections due to granulocyte deficiency, including bacterial infection, fungal infection, and viral infection. If poorly controlled, it is easy to lead to sepsis, which will lead to a worse prognosis and irretrievable outcome.

The oxidative stress after hematopoietic stem cell transplantation is in a delicate balance, and its specific mechanism remains to be elucidated. Based on this, we conducted a literature search and a novel scientometrics research on this hot topic. The initial search was based on the topic of hematopoietic stem cell transplantation and oxidative stress. For the retrieved publications, we conducted cluster analysis on all the keywords and mapped the cooccurrence network. The purpose was to preliminarily get the relationship between the research hot spots under the relevant subjects, so as to select the hot information or topics related to them. In this research, in addition to “oxidative stress” as the search term, the words “progenitor cells”, “self-renewal”, “iron overload”, and so on appear more frequently. In addition, there are some words that have appeared in recent years although they are less frequent, such as diagnostic impact, ineffectiveness, indoleamine 2,3-dioxygenase, and labile plasma iron. These words may become new hot spots in future applications or research. Readers can choose the keywords they are interested in or further explore the keywords that have a strong cooccurrence relationship with them and have a relatively recent appearance.

Next, in order to obtain more accurate results, we excluded studies unrelated to complications or prognosis, and only 59 publications were retained. Timeline view is used here to present the clustering results of keywords and their cooccurrence relationships. We also divided the keywords into 13 clusters, and each cluster is labeled with a tag. This is a summary of the keywords in this cluster. Through such tags, readers can quickly identify the hot spots they are interested in. For example, the tag of the first cluster is T cell, and the keywords related to immune cells or immunity are mainly presented in this cluster. In addition, hot spots and cooccurrence networks are also shown in the figure. The cooperation relationship between the countries/organizations of the screened publications is shown in the study. The observation of visual images can help readers choose which countries or organizations have more in-depth research in this field. In-depth analyses of the journals from which the publications are sourced were done. We first conducted a multidimensional statistical analysis of the data of the journals from which the selected publications came, including the statistics of the total number of publications, the number of citations of all publications, and the presentation of the value of different journals. We found that journals such as *Blood*, *Bone Marrow Translation*, and *Biology of Blood and Marrow Transplantation* have a high number of publications and citations and have considerable influence factors; readers can selectively read the publications from these journals based on the information when making inquiries. We also conducted cluster analysis based on the cocitation relationship for the source journals of the cited references. We believe that the cocitation relationship can effectively reflect the relevance and theme consistency between journals. For example, *Blood*, *Frontiers in Immunology*, and *Nature Reviews Endocrinology* appear in the same cluster. Perhaps this relationship can be used to select journals and help readers to contribute. In addition, we also presented the citation relationship between the publication source journals and their reference source journals and also perform simple clustering based on this. The specific situation has been marked with different labels. Meanwhile, an intensive reading part of recently published literatures was shown in Table 2 with appropriate and detailed descriptions [19–22].

As a negative effect of free radicals in the body, the process of oxidative stress is often and considered to be an important factor leading to diseases [23]. In particular, uncontrolled oxidative stress is one of the important culprits of HSCT and its related sepsis. Patients receiving HSCT may experience significant changes in the process of oxidative stress due to their potential malignant tumors and exposure to a wide range of chemotherapy and systemic antibiotics and further trigger chain reactions, such as cell and tissue damage, imbalance of gut microbiota, and production of harmful metabolites [24–26]. All of them can have direct or indirect effects on the post-HSCT operation. However, the precise mechanism of oxidative stress in HSCT complications and its related sepsis has not been fully elucidated. The occurrence of oxidative stress may be related to graft-versus-host disease or infection after HSCT, especially the systemic immune response. The main mechanisms are the respiratory burst of macrophages caused by infection or rejection, or the release of a large number of reactive oxygen species from mitochondria and endoplasmic reticulum after endothelial cell injury. Then, excessive reactive oxygen species will further stimulate macrophages and endothelial cells and form positive feedback. In addition, the depletion of the antioxidant system is also an important factor leading to the imbalance of oxidative stress. Many problems, such as how macrophages produce ROS during this process and the specific roles of different cytokines as well as the finding of biomarkers related to oxidative stress to predict prognosis, still need to be further explored. Meanwhile, the limitation of this research such as lacking more data on clinical trials as well as a comprehensive evaluation of the importance of clinical nursing and other objective factors on the successful treatment of HSCT still needs further research.

## 5. Conclusions

In this study, we conducted a novel scientometrics analysis on the publications on the topic of oxidative stress and HSCT. As far as keywords, countries, research institutions, and journals, we present their potential relationships, including cooccurrence relationships, coauthor relationships, and cocited relationships, in a variety of analytical ways. The multidimensional visual analysis helps readers to select the hot spot information they are interested in according to the chart, so as to carry out further research, clarify the mechanism, and optimize the clinical diagnosis and treatment measures.

## Figures and Tables

**Table 1:** The keywords with the top 20 number of occurrences.

Keyword	Occurrences	Total link strength
oxidative stress	189	839
self-renewal	53	327
transplantation	52	248
bone-marrow	50	242
progenitor cells	38	188
mice	33	154
bone-marrow-transplantation	30	128
hematopoietic stem cells	28	164
expression	26	139
hematopoietic stem-cells	26	86
stem-cells	26	118
in-vivo	24	137
inflammation	24	80
differentiation	20	106
stem-cell transplantation	20	91
activation	19	91
life-span	19	109
hematopoietic stem cell transplantation	18	73
proliferation	18	105
mouse model	17	73
apoptosis	16	90
DNA-damage	15	83
disease	14	69
hematopoietic stem	14	69
in-vitro	14	83
iron overload	14	80
aging	13	74
versus-host-disease	12	58
quiescence	11	64
therapy	11	55

**Table 2:** The multidimensional presentation and intensive reading of recently published literatures.

Author	Journal/IF/year	Study object	Treatment	Result	Conclusion
Patterson et al.	*Stem Cell Rev Rep* IF:6.692/2021	Patients with multiple myeloma	Patients received meloxicam with filgrastim before apheresis.	The number of CD34+ cells collected decreased significantly, the expression of CXCR4 on CD34+ cells decreased, and the proportion of CD4+/CD8+ T cells increased. RNA sequencing showed downregulation of oxidative phosphorylation related genes.	Mitigate hematopoietic stem and progenitor cells oxidative stress. Lessen stem cell exhaustion and enhance graft quality.
Rodionov et al.	*Bone Marrow Transplant* IF:5.174/2022	G-CSF mobilized human peripheral blood cells and nonobese diabetic-severe combined immune deficiency (NOD-SCID) IL2R*γ*null (NSG) mice.	A treatment of MPBCs with Fas ligand (FasL, CD95L).	Selectively induce apoptosis of CD3+ T cells, B cells, and antigen presenting cells, but CD34+ hematopoietic stem cells and progenitors. Reduce IFN-*γ* secreted by cells.	Increase the possibility of graft survival and function, reduce GVHD, and reduce the proinflammatory capacity of macrophages.
Wang et al.	*Int Immunopharmacol* IF:5.714/2020	BALB/c (H-2d) mice induced GVHD.	Treated with the combination of BBR and CsA.	Reduce weight loss and GVHD index scores. Reduce intestinal and liver damage, inflammation, and oxidative stress. Suppress NF-*κ*B signaling in liver and intestine. Reduce the number of Th1 cells.	Reduce oxidative stress and alleviate inflammatory response induced by acute GVHD. Improve the survival rate of GVHD mice.
Rezende et al.	*J Immunol Res* IF:*4.493*/2019	C57BL/6 and B6D2F1 mice induced GVHD.	Intraperitoneally inject apocynin during the experiments.	The treated mice reduced mortality and disease progression, reduced oxidative stress, reduced liver and intestinal damage, and inhibited inflammation.	Regulate the inflammatory response related to GVHD without impairing the engraftment which is associated with controlling oxidative stress.

## Data Availability

The original contributions presented in the study are included in the article; further inquiries can be directed to the corresponding authors.
